# Two-colour interferometry and switching through optomechanical dark mode excitation

**DOI:** 10.1038/s41467-020-15625-x

**Published:** 2020-05-05

**Authors:** David P. Lake, Matthew Mitchell, Barry C. Sanders, Paul E. Barclay

**Affiliations:** Department of Physics and Astronomy and Institute for Quantum Science and Technology, University of Calgary, Calgary, AB T2N 1N4 Canada

**Keywords:** Microresonators, Micro-optics, Optomechanics

## Abstract

Efficient switching and routing of photons of different wavelengths is a requirement for realizing a quantum internet. Multimode optomechanical systems can solve this technological challenge and enable studies of fundamental science involving widely separated wavelengths that are inaccessible to single-mode optomechanical systems. To this end, we demonstrate interference between two optomechanically induced transparency processes in a diamond on-chip cavity. This system allows us to directly observe the dynamics of an optomechanical dark mode that interferes photons at different wavelengths via their mutual coupling to a common mechanical resonance. This dark mode does not transfer energy to the dissipative mechanical reservoir and is predicted to enable quantum information processing applications that are insensitive to mechanical decoherence. Control of the dark mode is also utilized to demonstrate all-optical, two-colour switching and interference with light separated by over 5 THz in frequency.

## Abstract (web summary)

Efficient switching and routing of photons of different wavelengths is desirable for future quantum information applications. To this end the authors demonstrate interference in a multimode system between two optomechanically induced transparency processes in a diamond on-chip cavity.

## Introduction

Interference is a ubiquitous physical phenomenon central to applications ranging from detection of gravitational waves^1^ to the implementation of modulators and other integrated photonics technology^2–4^. Each of these examples interfere light at, or near, the same wavelength to convert differences in phase to changes in intensity. The emergence of frequency bin qubits^5–7^ and a desire to interface quantum networking components based on different photonic technologies^8^ have created the need for devices that interfere light with widely separated frequencies. Typically, this challenge has been addressed using non-linear atomic^9,10^ or solid-state^11–14^ materials, whose non-linear optical susceptibility combined with precise photonic dispersion engineering can mediate interactions between different wavelengths of light. Here we demonstrate that cavity optomechanics^15^ provides a realization of multi-colour optical interference that can be implemented in transparent linear materials through light’s interaction with a nanofabricated mechanical resonator. This process does not suffer from saturation given the simple harmonic oscillator nature of our excitations, and can be realized with effective cooperativity exceeding unity.

By coherently coupling light confined in an optical cavity to the motion of a mechanical resonance of the same cavity, light can be slowed and stored^16–18^. The signature of this coherent optomechanical coupling between phonons and photons is a narrow transmission window in the otherwise opaque optical cavity resonance spectrum, referred to as optomechanically induced transparency (OMIT)^16,17^, whose highly dispersive optical response is independent of the phase of its input field. However, phase can be critically important to the optical properties of multimode optomechanical systems, in which multiple optical fields are injected into an optomechanical device. In the optical domain, multimode cavity optomechanical devices have been used for experimental demonstrations of wavelength conversion^19–21^. Microwave-frequency multimode devices have enabled low-noise frequency conversion^22^ and entanglement between photons^23^, whereas hybrid electro-optomechanical devices have used coherent interference between optomechanically and piezomechanically driven motion to bridge microwave and optical frequencies^24^. Optical-frequency optomechanical devices whose intensity response is sensitive to the relative phase of multiple input optical fields with widely separated wavelengths, i.e., that involve interference between different colours of light, have yet to be reported despite proof-of-principle demonstrations based on atomic media^25–28^ and recent advances in cavity optomechanical mediated coupling between multiple mechanical modes^29,30^.

Here we utilize a cavity optomechanical device with two optical modes coherently coupled to a single mechanical resonance to show that double OMIT (DOMIT), in which two optical modes coherently couple to the same mechanical mode, enables the interference between photons separated by over 5 THz in frequency. Exploiting this effect, we demonstrate a phase-sensitive two-colour optical XOR gate^31^. This multi-colour interference is mediated by the mechanical bright mode and the mechanical dark mode^20,32^, which are directly excited for the first time here. This builds on previous studies using multimode optomechanical systems, which achieved partial dark mode excitation. In these works, which were focused on wavelength conversion and thus involved a single probe, finite perfect dark mode excitation can only be realized in the limit of infinite optomechanical cooperativity, *C*^20^. The optical XOR gate demonstrated here will be useful for multi-colour classical and quantum optical information processing, e.g., for demonstrating quantum interference^12,13^ between frequency binned qubits^5–7^, as well as optical sensing and metrology^14^ using interference between widely separated wavelengths.

## Results

### Double optomechanically induced transparency (DOMIT)

**Fig. 1:**
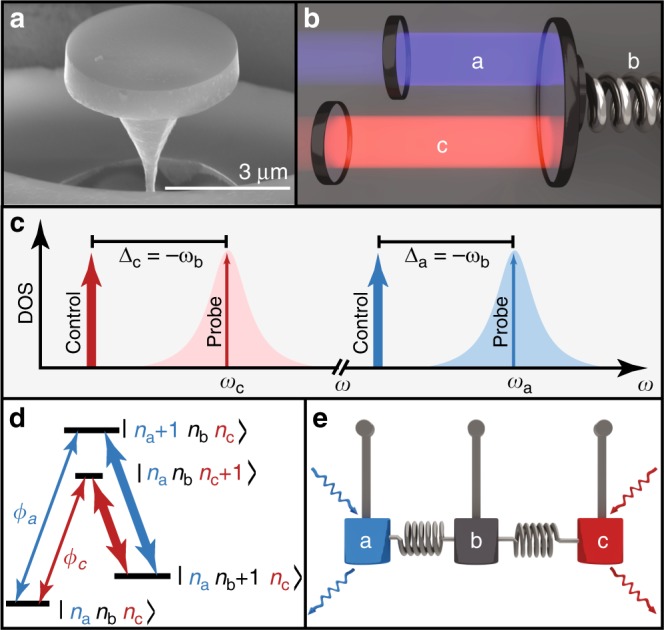
Multimode optomechanical system coupling two optical modes to a mechanical mode of a diamond microdisk. **a** Scanning electron micrograph of a diamond microdisk similar to the ~5 μm diameter one studied in this work. **b** Schematic of a multimode optomechanical system where two Fabry Perot optical modes, *a* and *c*, are coupled to the same mechanical mode, *b*. **c** Frequency-domain illustration of the control and probe lasers used throughout this work, and their respective optical cavity modes. **d** The double-Λ level scheme used in this work, where the labels *n*_*a*_, *n*_*b*_, *n*_*c*_ represent the energy levels of modes *a*, *b* and *c*, respectively. The thick lines represent photon–phonon exchange mediated by the control field, and the thin lines represent the probe fields with phase difference *ϕ* = *ϕ*_*a*_ − *ϕ*_*c*_. **e** DOMIT can be described by three coupled harmonic oscillators. The optical modes may be driven in-phase-, or out-of-phase, exciting either the mechanically bright or the mechanically dark mode.

Photon-photon interference and mechanical dark mode excitation demonstrated here uses a diamond microdisk cavity optomechanical system (Fig. 1a) whose essential elements can be analyzed as a generic Fabry Perot cavity shown in Fig. 1b. The cavity supports two optical modes with widely separated frequencies *ω*_*a*_ and *ω*_*c*_, each coupled via radiation pressure to a common mechanical resonator with frequency *ω*_*b*_ established in the generic system by one of the cavity mirrors. Each optical mode is excited with a weak probe field and a strong control laser that is red-detuned from its cavity mode by the mechanical frequency, Δ_*a*_ = Δ_*c*_ = −*ω*_*b*_, represented graphically in Fig. 1c. If the mechanical frequency exceeds the dissipation rate of each optical cavity mode, the system is in the resolved sideband regime, and the interaction Hamiltonian simplifies to^15^:

1 $$\hat H_{{\mathrm{int}}} = - \hbar \left[ {\left( {G_{a}\hat a^\dagger + G_{c}\hat c^\dagger } \right)\hat b + \left( {G_{a}^ \ast \hat a + G_{c}^ \ast \hat c} \right)\hat b^\dagger } \right],$$

where $\hat a(\hat a^\dagger )$ and $\hat c(\hat c^\dagger )$ are the creation (annihilation) operators of the optical probe field photons, $\hat b(\hat b^\dagger )$ is the creation (annihilation) operator of the mechanical resonator phonons, and we employ the rotating wave approximation. Here *G*_*a*_ = *g*_*a*_*α*_*a*_ and *G*_c_ = *g*_*c*_*α*_*c*_ are the control-field assisted optomechanical coupling rates for modes *a* and *c*, set by the single photon–phonon coupling rates *g*_*a*,*c*_, and the control-field amplitudes $\left| {\alpha _{{a},{c}}} \right|^2 = n_{{a},{c}}$. The specific cavity geometry determines *g*_*a*,*c*_, which generally increases as the effective cavity length decreases, whereas the control-field amplitudes are set by their intracavity photon numbers *n*_*a*,*c*_, which are typically limited by non-linear optical effects in the cavity. The system can be described by a double-Λ energy diagram, as illustrated in Fig. 1d, which forms a closed loop under excitation from the two sets of control and probe fields.

In this DOMIT configuration, each of $\hat a$, $\hat b$ and $\hat c$ oscillate in the rotating frame at the same frequency. This allows relative phases between the various fields to be defined, despite their typically vast frequency differences. The key property is that the phases of each of the four fields forming the double-Λ loop affect the optical response^33,34^. This is in contrast to a single-Λ system, whose optical response depends only on the intensity of the control field. This behaviour is clearly elucidated by studying symmetric and antisymmetric combinations of the cavity’s optical modes. These modes are referred to as the ‘mechanically dark’ $( {\hat \zeta _{{\mathrm{dk}}} = (G_{c}\hat a - G_{a}\hat c)/({\mathrm{i}}\bar G)} )$ and ‘mechanically bright’ $( {\hat \zeta _{{\mathrm{br}}}} = {(G_{a}^ \ast \hat a + G_{c}^ \ast \hat c)/(\bar G)} )$ modes, as the dark mode can be entirely decoupled from the mechanical resonator, while the bright mode can be maximally coupled; here $\bar G = \sqrt {\left| {G_{a}} \right|^2 + \left| {G_{c}} \right|^2}$.

These three modes are analogous to the modes of three coupled pendula, as shown in Fig. 1e, in which the outer two pendula move in opposite directions, while the central pendulum is stationary (dark mode); alternatively the three pendula move in the same direction (bright mode). This basis, with analogies in atomic physics^25,28^, elegantly reveals the importance of optical phase to the system’s behaviour. The classical amplitudes of the bright and dark modes when both probe fields are resonant with their respective cavity modes are (see Supplementary Note 4):

2 $$\zeta _{{\mathrm{dk}}} = \frac{{2\sqrt {\kappa _{{\mathrm{ex}}}} s_{{\mathrm{in}}}}}{\kappa }{\mathrm{sin}}\left( {\phi /2} \right),$$

3 $$\zeta _{{\mathrm{br}}} = \frac{{2\sqrt {\kappa _{{\mathrm{ex}}}} s_{{\mathrm{in}}}}}{{\kappa \left( {1 + \bar C} \right)}}{\mathrm{cos}}\left( {\phi /2} \right),$$

where *κ*_ex_ and *κ* are the external coupling and total loss rates of the cavity modes, respectively, *s*_in_ is the input amplitude of the two probe lasers, $\bar C = 4\bar G^2/\kappa \gamma _{b}$ is the two-mode optomechanical cooperativity, and *γ*_*b*_ is the damping rate of the mechanical resonance. For simplicity, we assume that *κ*, *κ*_ex_ are the same for each optical mode, and set *s*_in_ to be equal to reflect the fact that we balanced the input probe field amplitudes in the experiment. In our experimental setup described below, *ϕ*_*a*_ is the phase difference between control and probe fields input to mode *a* and *ϕ*_*c*_ is the phase difference between control and probe fields input to mode *c*. The total phase difference is then *ϕ* = *ϕ*_*a*_ − *ϕ*_*c*_. As each of the probe fields are derived from their respective control field via electro-optic modulation in our experiment, changes to the control field phases do not affect the system response because they will also shift the probe phase by the same amount. Note that in principle the system could be operated with any of the four controls and probe phases used to determine the nature of the interference.

Equations (2–3) show that adjusting *ϕ* to ±*π*, e.g., by delaying one of the probes, allows complete selective excitation of *ζ*_dk_ without requiring $\bar C \to \infty$. When all DOMIT processes are resonant, this delay corresponds to a half period of the mechanical resonator. Equations (2–3) also show that full optical power transfer to the dark mode is possible, while the bright mode has a reduced maximum amplitude due to coherent optomechanical transfer of energy to the mechanical resonator. Optically manipulating the system in this basis is central to optomechanical wavelength conversion free from mechanical thermal decoherence effects^32,35^ when the optomechanical coupling exceeds the thermal decoherence rate^19,20,36^. However, none of these previous single OMIT studies have completely isolated or selectively populated the mechanically dark mode. Conceptually related studies demonstrating optomechanical control of interference between two mechanical modes^30^ have not yet been used to interfere different colours of light.

To demonstrate DOMIT, we evanescently coupled control and probe fields via an optical fibre taper waveguide into modes of a diamond microdisk device similar to that in Fig. 1a and previous studies^37,38^. As shown in Supplementary Fig. 1, our modes have resonant wavelengths *λ*_*a*_ ~ 1520 nm and *λ*_*c*_ ~ 1560 nm and sufficiently low optical loss (*κ*_*a*_/2*π* ~ 0.87 GHz, *κ*_*c*_/2*π* ~ 1.20 GHz) to allow resolved sideband optomechanical coupling to the microdisk’s *ω*_*b*_/2*π* = 2.1 GHz fundamental mechanical radial breathing mode (dissipation rate *γ*_*b*_/2*π* = 0.285 MHz). Typical optical and mechanical mode spectroscopy measurements are described in Supplementary Note 1. The per-photon optomechanical coupling rates, *g*_*a*_ = *g*_*c*_ ~ 2*π* × 25 kHz, allow optomechanical cooperativity >1 and observation of OMIT when approximately *n*_*a*,*b*_ > 5 × 10^5^ control photons are coupled into either of the cavity modes^38^. Achieving this large photon number is possible in our microdisks due to diamond’s low non-linear absorption and excellent thermal properties. In all of our measurements presented below, the input control fields are detuned from their respective cavity modes such that OMIT conditions Δ_*a*_ = Δ_*c*_ = −*ω*_*b*_ for each mode are satisfied. Note that the microdisks support regularly spaced modes spanning the IR and visible spectrum^37^; our modes are chosen due to the compatibility of their wavelengths with telecommunications equipment needed for the measurements described below.

**Fig. 2:**
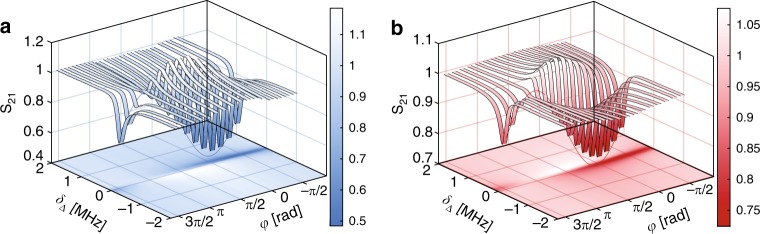
Measurement of double optomechanically induced transparency. **a**, **b** Cavity optical response to the probe signal for modes *a* and *c*, respectively, as a function of cavity-probe detuning *δ*_Δ_ and the probe phase difference *ϕ*, as recorded by the vector network analyzer from the photodetected heterodyne signal. The control fields are at fixed detuning Δ_*a*_ = Δ_*c*_ = −*ω*_*b*_.

To excite *ζ*_dk_ and *ζ*_br_, OMIT spectra^38^ for modes *a* and *c* were recorded for varying relative phase *ϕ* between the two probe fields, as shown in Fig. 2a, b. In these measurements, *ϕ* is controlled by adjusting a delay between two radio frequency (RF) signals split from the same signal generator, which are then used to create the probe fields through optical modulation of two independently running control lasers as shown in Supplementary Fig. 2 (see ‘Methods’ and Supplementary Note 1 for details). Our setup is robust to phase drifts of either control laser, as discussed above. Each pair of probe and control fields is isolated by optical filtering and then detected on a high-speed photodetector as a function of varying probe-cavity detuning *δ*_Δ_. Each probe amplitude is then measured by downmixing the heterodyne signal that it creates through interference with its corresponding control field using a vector network analyzer. At *ϕ* = 0, the deep OMIT window present in each probe output spectrum when *δ*_Δ_ = 0 indicates excitation of *ζ*_br_, whereas, when *ϕ* = *π*, the response of the bare cavity, which is broad compared with the OMIT window (*κ* ≫ *γ*_*b*_ (1 + *C*)), is restored for all probe detunings, indicating excitation of *ζ*_dk_. The depth of the OMIT window as a function of the phase delay is fit using solutions to Eqs. (2–3) in the *a* and *c* basis (see Supplementary Note 4), as is shown for $\delta _{\mathrm{\Delta }}^{{a},{c}} = 0$ in Fig. 2a, b, where it is seen to agree well with theory. The OMIT line-shapes of each mode deviate from a Lorentzian when studied individually^16,17^ due to the use of an amplitude modulator with non-zero chirp (see Supplementary Note 2 and Supplementary Fig. 3) in generating one of the probe lasers, and imperfect detuning of the control fields, as analyzed in previous work^39^. Differences in the Fano phase of each OMIT line-shape then results in constructive or destructive interference features for non-zero *δ*_Δ_ visible in Fig. 2a, b.

### Optomechanical bright and dark mode coupling

When OMIT resonance conditions for both modes are satisfied, i.e., Δ_*a*_ = Δ_*c*_ = − *ω*_*b*_ and $\delta _{\mathrm{\Delta }}^{a} = \delta _{\mathrm{\Delta }}^{c} = 0$, the *ζ*_br_ and *ζ*_dk_ states are decoupled from each other. However, as shown in Supplementary Fig. 5, by incrementing cavity-probe detuning of one mode by +*δ*_Δ_, and decrementing cavity-probe detuning of the other mode by −*δ*_Δ_, we induce a bright–dark state coupling. This can also be accomplished by shifting Δ_*a*_ and Δ_*c*_ in the same manner. Coupling between bright and dark states manifests as a temporal oscillation in the intensity of the probe fields transmitted through the cavity, allowing differences in their interaction with the dissipative mechanical resonator to be observed directly (see Supplementary Note 3).

**Fig. 3:**
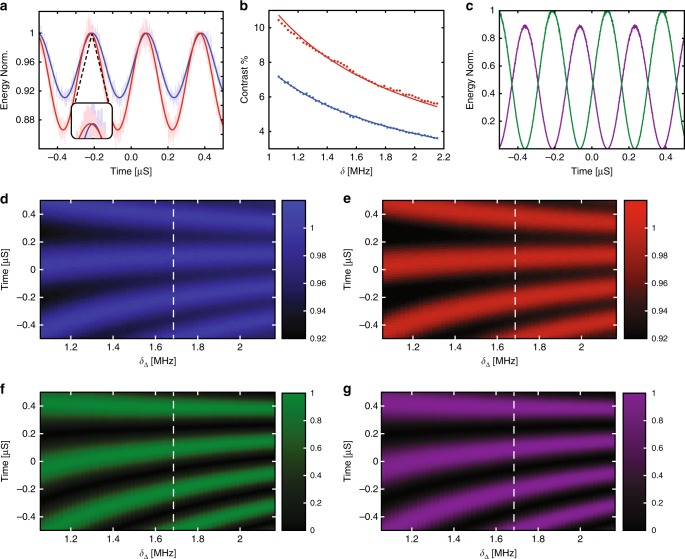
Measurement of coupling between the mechanically dark and bright modes. **a** Example of the oscillations in stored energy in mode *a* (blue trace) and mode *c* (red trace) for 2*δ*_Δ_ = 3.37 MHz, measured by downmixing the heterodyne signal at each colour in the time domain to isolate each probe amplitude. Inset: highlight of the slight time delay between the output of modes *a* and *c* caused by dispersion in the setup. **b** Amplitude of the oscillations for mode *a* (blue) and mode *c* (red) as a function of *δ*_Δ_, and corresponding predictions from the model given in the text. **c** Normalized bright (purple) and dark (green) state energy for 2*δ*_Δ_ = 3.37 MHz, as inferred from the optical output of modes *a* and *c* shown in **a**. **d**–**g** Oscillations as a function of time and *δ*_Δ_ of outputs from modes *a*, *c*, *ζ*_br_ and *ζ*_dk_, respectively.

Our measurement of both probe colours is plotted in Fig. 3a, for the case that 2*δ*_Δ_ = 3.37 MHz and *ϕ* = 0, after digitally downmixing the total (probe and control) photodetected signal from each colour recorded on a high-speed oscilloscope to remove fast oscillations near *ω*_*b*_ from beating between probe and control fields. Each downmixed signal is proportional to the amplitude, or equivalently, the square root of energy of the intracavity field at its respective probe frequency. As the modulation depth is bounded above by the dark-state transmission (bare cavity response), and bounded below by the bright-state transmission (OMIT window depth), the oscillation amplitude follows the *δ*_Δ_ dependence of the OMIT features. This dependence is confirmed by measuring the dependence of oscillation amplitude on increasing *δ*_Δ_, as shown in Fig. 3b, which matches well with theoretical predictions (see Supplementary Notes 5 and 6 for details), giving estimates of $\bar C = 3.6$ and $\bar C = 4.2$ from fits to the *ω*_*a*_ and *ω*_*c*_ photodetected signals, respectively. This difference in the cooperativities is attributed to non-ideal modulation when creating each probe and imbalance of the cooperativities of each mode.

We can also reconstruct the output of *ζ*_dk_ and *ζ*_br_ by inferring *a* and *c* from the measured phase and amplitude of the output probe fields in Fig. 3a. This reconstruction requires the additional step of accounting for a slight phase shift due to differing optical path lengths at *ω*_*a*_ and *ω*_*c*_ caused by dispersion in the experimental setup, whose effect can be seen in the inset of Fig. 3a. This deleterious phase shift was corrected in post-processing while determining *ζ*_br_ and *ζ*_dk_. An example of the reconstructed bright and dark mode intensity is shown in Fig. 3c, where flopping between the bright and dark states is evident. Notably, a difference in maximum intensity of *ζ*_dk_ and *ζ*_br_ is evident from their differing peak values. This difference can be related to the energy dissipated by the bright state due to its interaction with the mechanical resonance, and is found from Eqs. (2–3) generalized for non-zero *δ*_Δ_ (see Supplementary Notes 5 and 6) to scale as $1 + \bar C/\left( {1 + 4\left( {\delta _{\mathrm{\Delta }}/\gamma _{b}} \right)^2} \right)^{ - 2}$. Additional measurements of the intensity of *a*, *c*, *ζ*_br_ and *ζ*_dk_ as a function of *δ*_Δ_ and time are plotted in Fig. 3d–g, which clearly show how the oscillation period decreases with increasing *δ*_Δ_, as expected theoretically. The effect of mismatch in operating parameters between mode *a* and *c* is calculated in Supplementary Note 5 and Supplementary Fig. 4, where the main effect of mismatch in optomechanical coupling or probe amplitudes is to decrease the contrast of the interference, whereas mismatch in frequency or optical decay rates leads to dissipative and dispersive coupling between the bright and dark states.

### Two-colour switching

The phase-dependent response of this multi-colour DOMIT system, together with our ability to selectively excite *ζ*_dk_ or *ζ*_br_ can be harnessed to create a phase-dependent all-optical switch. In this device, the output intensity of one probe is dependent on the phase of the other probe, and follows the truth table of an XOR gate with probe field phases of 0 and *π* mapping onto Boolean values 0 and 1. The maximum contrast achievable is determined by the maximum OMIT dip depth, and is given by $\bar C^{2}/(1 + \bar C)^{2}$. This indicates that, in principle, the contrast can be made to approach unity for systems with large cooperativity. The main advantage of the switching scheme demonstrated here compared with reference^30^ is that the switch inputs and outputs are all-optical, and the scale of frequency differences in our systems is large, i.e. the optical modes in our system are separated by 5 THz, compared with only 180 kHz of the nearly degenerate mechanical modes in reference^30^.

**Fig. 4:**
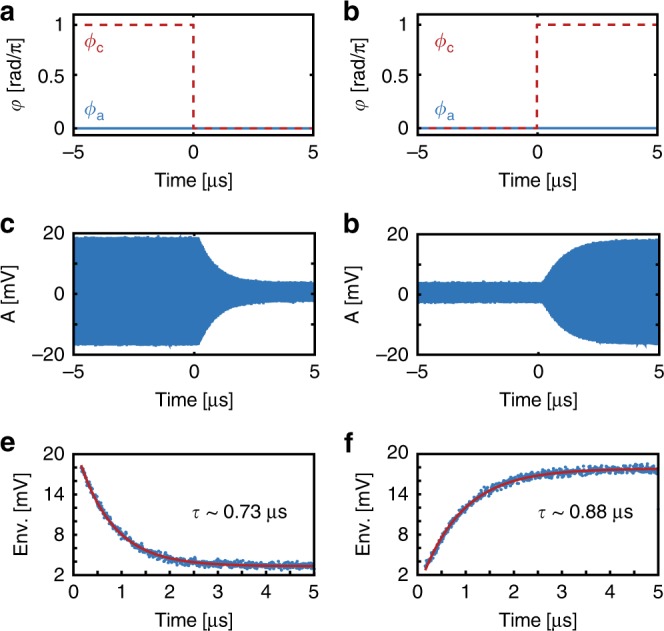
Demonstration of a phase-dependent all-optical switch. **a**, **b** Phases of the input probe fields vs. time which cause probe *c* to switch the response of probe *a* off and on, respectively. **c**, **d** Photodetected cavity output of probe *a* when the input phase of probe *c* is switched temporally as shown in **a** and **b**. The rapid oscillations are caused by beating between the control and probe, and the envelope reveals the action of the switch. **e**, **f** Envelope extracted from the peak values of the data in **c**, **d** and fit with exponentially falling and rising functions, respectively.

This switching action can be inferred from Fig. 2a, b, and directly observed in the time domain by varying the phase *ϕ*_*c*_ of the mode *c* probe following a temporal step function, while maintaining constant phase *ϕ*_*a*_ of the mode *a* probe, as sketched in Fig. 4a for switching off and Fig. 4b for switching on. Experimental time domain data showing the resulting change in probe *a* transmission is shown in Fig. 4c, d. As mentioned above, the relative phase *ϕ* = *ϕ*_*a*_ − *ϕ*_*c*_ is controlled by introducing an electrical phase delay in the RF signal driving the electro-optic modulator responsible for creating the mode *c* probe (see Methods and Supplementary Note 1 for details). The rapid oscillations are due to beating between the probe and control laser, whereas the oscillation envelope is proportional to the amplitude of the transmitted probe. From this upper envelope, shown in Fig. 4e, f, we can measure the switch response speeds: fits with an exponential function yield fall and rise times of 0.73 and 0.88 μs, respectively. Assuming that *κ*_*a*,*c*_ ≫ *γ*_*b*_, the switching speed can be shown to be $\tau = 1/\gamma _{b}(1 + \bar C)$ (see Supplementary Note 7), which approaches 0 for sufficiently large $\bar C$, enhancing the switching speed beyond 1/*γ*_*b*_. This switching speed implies $\bar C = 2.96$ and $\bar C = 3.89$, respectively, for the fall and rise times illustrated in Fig. 4c, d, consistent with expectations from the control-field amplitudes. The observed differences in the fall and rise times are presumed to be due to differences in intracavity control-field amplitudes during the respective measurements. Larger contrast, which would be beneficial for switching applications, could be realized by further increasing *C*. Single photon operation would require removal of thermal phonons, either through cryogenic cooling or higher cooperativity combined with feedforward noise suppression^40^.

## Discussion

In summary, we have demonstrated coherent interference between spectrally-separated optical modes mediated by optomechanical coupling. By adjusting the phase between different colour probe fields entering the cavity, we selectively excite either a mechanically bright or a mechanically dark mode, and demonstrate controllable coupling between the two modes. Notably, we exploit the difference between bright- and dark-state transmission to demonstrate a two-colour, all-optical switch, where the control and target are at different wavelengths.

Our system has great potential for applications to quantum information processing where interference between frequency binned qubits is desirable^6^, such as frequency-domain Hong–Ou–Mandel interference^12,13^, chromatic and time-domain interferometry^5,14,41^ and microwave-to-optical conversion via the optomechanical dark mode^24^. Furthermore, as detailed in Supplementary Note 8, our technique could be extended to many optical modes whose operating wavelengths are only limited by the transparency of diamond and the existence of high-*Q* modes; thus, our technique could lead to many-colour interference processes. Finally, we note that this interference is quite general and could be utilized for non-optical inputs, such as magnetic or electric fields, provided they couple to the mechanical degree of freedom^42^. Future experiments operating in the quantum domain will benefit from cryogenic pre-cooling of the device to its mechanical quantum ground state, which is achievable at dilution fridge temperatures for the >2 GHz frequency mechanical mode measured in this article.

## Methods

### Device

The device used for all experiments in this work is a ~5 μm microdisk resonator fabricated from single crystal diamond using the process outlined in references^43,44^. A full schematic of the experimental apparatus used in the experiments is given in Supplementary Note 1. In all of the measurements light was coupled evanescently into and out of the microdisk using a dimpled optical fibre taper positioned using motorized stages, as discussed in references^37,38^.

### Measurement of DOMIT

To measure the OMIT spectra of each microdisk mode, sidebands were created on each respective control laser for use as probe fields, using either phase, *ϕ*(*t*), or amplitude, *A*(*t*), electro-optic modulators. For the data in Fig. 2, the electrical RF drive for each modulator was derived from the same vector network analyzer, with one path undergoing a controllable phase shift relative to the other. This controllable phase shift was achieved by placing an electronic phase shifter before one of the electro-optic modulators. Because the phase shifter transmission varied as a function of phase, a variable electrical attenuator was calibrated and used to maintain balance between the probe laser powers at every step in phase, by controlling the RF modulation amplitude. An optical variable attenuator was used on the 1560 nm laser arm to attempt to balance the input power of each laser before they were combined via a 50/50 waveguide coupler, and amplified with an erbium doped amplifier before being routed to the sample chamber (a nitrogen purged enclosure) and device. The other output of the 50/50 coupler was used to perform slow laser wavelength locking via a photodetector and optical spectrum analyzer connected to the control computer. The signal exiting the sample chamber was then divided on a 90/10 waveguide coupler. The 10% port was routed to a low speed photodetector for use in measuring the cavity transmission during the initial setup, and the 90% port was sent to the wavelength division multiplexer (WDM). By connecting the WDM to a high-speed photodetector, the output of either mode *a* or *c* could be selected.

### Measurement of bright–dark mode coupling and switching

For the bright–dark mode coupling experiment (Fig. 3) and the time domain switch (Fig. 4), a two-channel arbitrary waveform generator (AWG) was used as the RF source, with one channel assigned to each modulator. Acquisition was performed using a digital spectrum analyzer (DSA), which was triggered by the AWG. To isolate the beat note between the probe field of one mode and the converted probe from the other mode the signal acquired on the DSA was digitally downmixed post acquisition.

## Supplementary information

Supplementary Information

Peer Review File

## Data Availability

The datasets generated during and/or analyzed during the current study are available from the corresponding author on reasonable request.
