# Supplementary material for: Two-colour interferometry and switching through optomechanical dark mode excitation
Source: Nat Commun. 2020 May 5;11:2208. doi: 10.1038/s41467-020-15625-x (PMC7200651; doi:10.1038/s41467-020-15625-x)
Supplement: Supplementary file 1 — Supplementary Information [file 41467_2020_15625_MOESM1_ESM.pdf]

Supplementary Information - Two-colour interferometry and switching through optomechanical dark mode excitation

Lake et al.

Supplementary Note 1 - Experiment setup and calibration

The optomechanical cavity utilized in this work is a single–crystal diamond (SCD) microdisk, fabricated according to the process outlined in Refs.[1,2], an example of which is shown in Supplementary Figure 1(a). An advantage of microdisk cavities is that they support multiple optical whispering gallery modes across their transparency window, all of which exhibit dispersive optomechanical coupling to the fundamental radial breathing mode (RBM) of the microdisk[3], as illustrated schematically in Supplementary Figure 1(b). Diamond's large electronic bandgap, Young's modulus, and best-in-class thermal conductivity make it an ideal material for use in cavity optomechanics as it can support large intracavity photon number N, and high optical and mechanical quality factors. Additionally, colour center qubits present in diamond, such as silicon and nitrogen vacancies, make it a promising platform for realizing hybrid quantum systems[4].

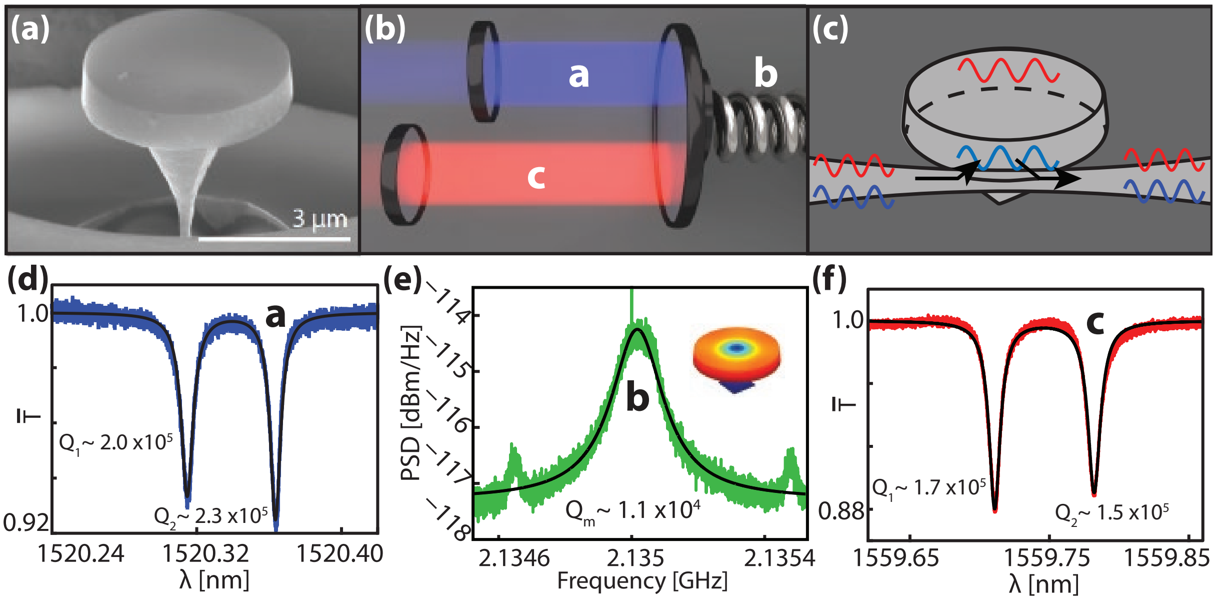

Supplementary Figure 1: Characterization of optical and mechanical modes of the diamond microdisk utilized in this work. (a) Scanning electron micrograph of a diamond microdisk similar to the one used in this work. (b) Cartoon of a canonical multimode optomechanical system. Here mode b, represented as a spring, is dispersively coupled to both optical modes a and c. (c) Cartoon of fiber taper–microdisk coupling. (d) Normalized fiber taper transmission scan of mode a used in this experiment, with fit. (e) Power spectral density of the fiber taper transmission when the input laser is tuned near a cavity mode, revealing fluctuations from thermomechanical motion of the cavity's mechanical radial breathing mode, b. A COMSOL simulated displacement field profile of the radial breathing mode is shown in the inset. (f) Normalized fiber taper transmission scan of optical mode c, with fit.

Light from two tunable diode lasers was coupled into and out of the microdisk using a dimpled optical fiber taper positioned adjacent to the microdisk as illustrated in the cartoon in Supplementary Figure 1(c). The spatial overlap of the evanescent field of the fiber and the optical modes of the microdisk permit efficient coupling, allowing measurement of cavity modes in transmission and reflection. Two telecommunications wavelength modes at $\lambda_\mathrm{a} = 2\pi c/\omega_\mathrm{a} = 1520$ nm and $\lambda_\mathrm{c} = 2\pi c/\omega_\mathrm{c} = 1560$ nm were selected for this work, as they were in the operating range of the available lasers (Newport TLB-6700) and optical amplifier (Pritel EDFA). However, this could be extended to visible wavelengths, where these devices have demonstrated high quality optical modes[5]. The optical modes are each dispersively coupled to the microdisk's fundamental mechanical radial breathing mode (RBM) whose frequency is $\omega_\mathrm{b}/2\pi = 2.1$ GHz, with vacuum optomechanical coupling rates, $g_{0,\mathrm{a}}, g_{0,\mathrm{c}} \sim 2\pi \times 25$ kHz. Measurements of the fiber taper optical transmission spectrum for wavelengths scanned across modes a and c are shown in Supplementary Figure 1(d,f), and the power spectral density of the fluctuations imparted on photodetected output due to thermally driven mechanical motion of the RBM when the input laser is tuned close to resonance with

an optical mode[6–8] is shown in Supplementary Figure 1(e). Note that both optical modes are standing wave doublets due to surface roughness induced coupling between the clockwise and counterclockwise propagating whispering gallery modes of the microdisks[9]. For all of the measurements presented here the long wavelength doublet mode was used, and the lower wavelength doublet mode was assumed to not influence the observed phenomena. This device operates in the sideband resolved regime, $\omega_\mathrm{b} \gg \kappa_\mathrm{a}, \kappa_\mathrm{c}$, where $\kappa_\mathrm{a}/2\pi \sim 0.87$ GHz, and $\kappa_\mathrm{c}/2\pi \sim 1.20$ GHz are the energy decay rates of the optical modes. A previous study[2] found that the optical quality factor, $Q = \omega/\kappa$, of this device is likely still limited by surface roughness induced by the fabrication procedure.

(a)

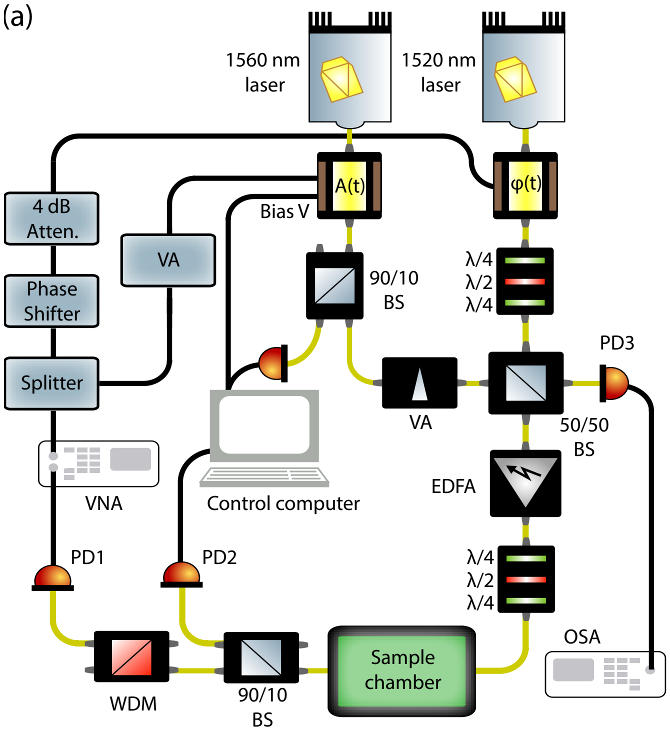

(b)

Parts & Equipment

Lasers - Newport TLB-6700
Phase shifter - Mini Circuits JSPHS-2484
Splitter - Mini Circuits ZFRSC-123-S+
Attenuator - Mini Circuits ZX73-2500-S+
VNA - Keysight E5063A
OSA - Ando AQ6317B
EDFA - Pritel LNHPFA-30-IO
A(t) & φ(t) - EOSpace PM-5S5-20-PFA-PFA-UV-UL, AZ-0K5-10-PFA-SFA
WDM - Montclair Optics MFT-MC-51-30 AFC/AFC-1
VA - Exfo FVA-3100
90/10 BS - Newport F-CPL-L22351-A
50/50 BS - Newport F-CPL-L22355-A
PD1 - Newfocus 1554-B
PD2 & PD3 - NewFocus 1621
AWG - Tektronix AWG70002A
DSA - Tektronix DSA70804B

Supplementary Figure 2: Apparatus used in this work, with corresponding equipment list. (a) Apparatus used in the experiments. The Vector Analyzer (VNA) acted as the RF source for modulators on each path. The resulting signal was spectrally filtered to isolate for the mode of interest. Key: OSA (Optical spectrum analyzer), BS (Beam splitter), VA (Variable attenuator), BS (Beam splitter), EDFA (Erbium doped fiber amplifier), WDM (Wavelength division multiplexer), PD (Photodetector). (b) Parts and equipment used in the experiment.

Supplementary Figure 2 shows a schematic representation of the experimental setup used for the above measurements and those shown in the main text. Additional information can be found in the methods section of the main text.

Supplementary Note 2 - Electro–optic modulation and probe measurement model

The results demonstrated here require strong control fields and weak probe fields, which were generated through EOM modulation of the control fields. Due to the available equipment, a phase EOM was used for the mode near ω_a and an amplitude EOM for the mode near ω_c. This leads to differences in the probe transduction as measured on the high speed PD.

For weak modulation ($\beta \ll 1$, where β is the index of modulation), we can assume the output of the EOM has three distinct frequency components at ω, $\omega \pm \omega_\mathrm{m}$, where ω is the frequency of the carrier tone and ω_m is the frequency of modulation. For convenience we will work in a frame rotating with the carrier at ω. The type of

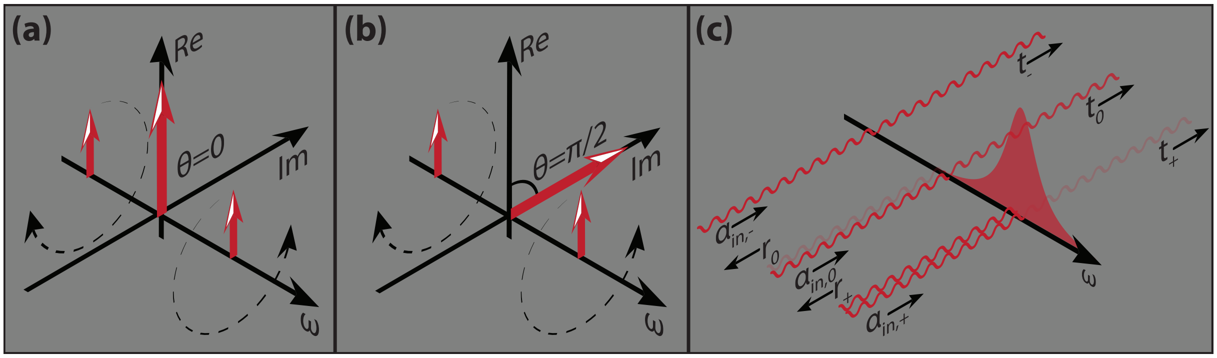

Supplementary Figure 3: Phasor illustration of amplitude and phase modulation, and expected cavity transmission and reflection for a modulated input field. (a) Frequency components of pure amplitude modulation in a frame rotating at the carrier frequency. The two sidebands are placed at $\pm\omega_{\mathrm{m}}$, where ω_{m} is the frequency of modulation. Here the modulation is in a direction parallel to the carrier tone. (b) Frequency components of a pure phase modulation. Unlike the case of amplitude modulation, the modulation occurs in a direction perpendicular to the carrier tone. (c) Illustration of the expected reflected and transmitted signals for the case of a red detuned pump laser modulated such that one sideband is near resonance with the cavity.

modulation can be inferred from the sum of the sideband components in the imaginary plane. For pure amplitude modulation they will oscillate parallel to the carrier with frequency ω_{m}, whereas for pure phase modulation they will oscillate perpendicular to the carrier as illustrated in Supplementary Figure 3(a,b). We note that since we are working in the rotating frame, we can choose the phase of the carrier out of convenience, as only the relative phase between the sidebands and the carrier tone influences the result.

In practice, the construction of amplitude EOMs is often such that the chirp is non-zero, which results in non-zero phase modulation of the outgoing field. With this in mind, we can write the transmission of the modulated field through the cavity as $|\alpha_{\mathrm{out}}\rangle = t\,|\alpha_{\mathrm{in}}\rangle$, where

$$t = \mathbf{diag}\{t_+, t_0, t_-\},\ |\alpha_{\mathrm{in}}\rangle = \alpha_{\mathrm{in}}^0 \left(\tfrac{\beta}{2} e^{-i\omega_m t}, e^{i\theta}, \tfrac{\beta}{2} e^{i\omega_m t}\right)^{\mathbf{T}}. \tag{1}$$

In the previous expression, t_+, t_0, t_- are the transmission coefficients at the upper sideband, carrier frequency, and lower sideband, respectively. The angle between the sidebands and the carrier is θ, where $\theta = n\pi$ for a pure amplitude modulator and $\theta = \pi/2 \pm n\pi$ for a pure phase modulator, where n is an integer.

The frequency components of the field transmitted through the cavity can be projected using the matrices

$$\mathbf{P}_+ = \begin{pmatrix} 0 & 1 & 0 \\ 0 & 0 & 1 \\ 0 & 0 & 0 \end{pmatrix},\ \mathbf{P_0} = \begin{pmatrix} 1 & 0 & 0 \\ 0 & 1 & 0 \\ 0 & 0 & 1 \end{pmatrix},\ \mathbf{P}_- = \begin{pmatrix} 0 & 0 & 0 \\ 1 & 0 & 0 \\ 0 & 1 & 0 \end{pmatrix}. \tag{2}$$

Using the above expressions, we can write the signal measured on the PD up to a constant as $S = S_0 + S_1 + S_2$, where

$$S_0 = \langle\alpha_{\mathrm{out}}|\,P_0\,|\alpha_{\mathrm{out}}\rangle, \tag{3}$$

$$S_1 \cos(\omega_m t + \phi_1) = \langle\alpha_{\mathrm{out}}|\,P_+\,|\alpha_{\mathrm{out}}\rangle + \langle\alpha_{\mathrm{out}}|\,P_-\,|\alpha_{\mathrm{out}}\rangle, \tag{4}$$

$$S_2 \cos(2\omega_m t + \phi_2) = \langle\alpha_{\mathrm{out}}|\,P_+^2\,|\alpha_{\mathrm{out}}\rangle + \langle\alpha_{\mathrm{out}}|\,P_-^2\,|\alpha_{\mathrm{out}}\rangle. \tag{5}$$

By electronic filtering we isolate the $\mathcal{O}(\omega_{\mathrm{m}})$ component of the signal, where

$$S_1 = \beta\left|t_0^* t_+ e^{-i\theta} + t_0 t_-^* e^{i\theta}\right|. \tag{6}$$

To find the expected signal we use $\theta = 0.6093$ [Rad], as measured directly from the OMIT spectra, and an agreement with the manufacturer's specifications. In this work the control laser is red detuned from a sideband-resolved cavity, as illustrated in Supplementary Figure 3(c). In this case the lower sideband passes un–attenuated

($t_- = 1$, $r_- = 0$), and the control laser is approximately real $t_0 \approx t_0^*$. In this case, to first order in modulation angle, the expected signal is

$$S_1 \approx \beta t_0 \sqrt{(r_+ - 1 - \cos(2\theta))^2 + \sin^2(2\theta)}, \tag{7}$$

where we have used the fact that $t_+ + r_+ = 1$. Using the chirp parameter[10] specified by the manufacturer for the amplitude EOM we calculate $\theta = 0.6093$ [rad]. This value also agrees well to direct fits to the OMIT lineshapes.

In the case of pure phase modulation ($\theta = \pi/2 \pm n\pi$) this simply reduces to

$$S_1 \approx \beta t_0 |r_+|. \tag{8}$$

Supplementary Note 3 - Data analysis

In order to examine the bright and dark state coupling as shown in Fig. 3 in the main text, time-domain data was directly acquired on the DSA. For this dataset we digitally down mixed by the carrier frequency ω_b, which allowed us to extract both the amplitude of the signal, and the phase relative to the carrier signal for modes a and c. Due to chirp in the amplitude modulator, dispersion in the fiber, and difference in the optical path length of the two output arms of the WDM, a delay between the mode outputs was observed. To correct for this we fit the oscillating output of each mode to a sinusoidal function, and subtract the phase difference. Using this we are able to reconstruct the output of the dark and bright states.

Supplementary Note 4 - Double optomechanically induced transparency

In this work, two optical modes a and c exhibit dispersive optomechanical coupling to the mechanical mode b. We denote the frequencies of these modes as ω_a, ω_c, and ω_b, respectively, and the vacuum optomechanical coupling rates as g_a, and g_c. This is modelled by the Hamiltonian $\hat{H} = \hat{H}_0 + \hat{H}_\mathrm{int}$, where $\hat{H}_0$ describes the internal dynamics of each mode and $\hat{H}_\mathrm{int}$ is the interaction Hamiltonian

$$\hat{H}_0 = \hbar\omega_\mathrm{a}\hat{a}^\dagger\hat{a} + \hbar\omega_\mathrm{b}\hat{b}^\dagger\hat{b} + \hbar\omega_\mathrm{c}\hat{c}^\dagger\hat{c}, \tag{9}$$

$$\hat{H}_\mathrm{int} = -\hbar g_\mathrm{a}\hat{a}^\dagger\hat{a}\left(\hat{b} + \hat{b}^\dagger\right) - \hbar g_\mathrm{c}\hat{c}^\dagger\hat{c}\left(\hat{b} + \hat{b}^\dagger\right). \tag{10}$$

We describe the coupling between the optical modes and a waveguide using input-output theory

$$\dot{\hat{a}} = \frac{\mathrm{i}}{\hbar}\left[\hat{H}, \hat{a}\right] - \frac{\kappa_\mathrm{a}}{2}\hat{a} + \sqrt{\kappa_\mathrm{a}^\mathrm{ex}}\hat{a}_\mathrm{in}, \tag{11}$$

$$\dot{\hat{c}} = \frac{\mathrm{i}}{\hbar}\left[\hat{H}, \hat{c}\right] - \frac{\kappa_\mathrm{c}}{2}\hat{c} + \sqrt{\kappa_\mathrm{c}^\mathrm{ex}}\hat{c}_\mathrm{in}, \tag{12}$$

where $\hat{a}_\mathrm{in}$ and $\hat{c}_\mathrm{in}$ are the input field operators for each optical mode, and κ_a, κ_c and $\kappa_\mathrm{a}^\mathrm{ex}$, $\kappa_\mathrm{c}^\mathrm{ex}$ are the total energy decay and waveguide–cavity coupling rates of mode a and c, respectively. Note that in this work the cavity is double-sided and consequently the cavity–waveguide coupling rate in each direction is $\kappa_\mathrm{ex}/2$.

For all scenarios described in this work control lasers were red-detuned from the cavity modes whereas probe lasers were tuned near resonance. Although the modulators create multiple sidebands, the spectral selectivity of the cavity is such that only one sideband will contribute to the physics of the problem. This allows us to linearize about the control fields using the substitutions $\hat{a} \to \alpha_\mathrm{a} + \hat{a}$, and $\hat{c} \to \alpha_\mathrm{c} + \hat{c}$, where $\alpha_\mathrm{a}, \alpha_\mathrm{c}$ are the classical control fields amplitudes, and $\hat{a}$, $\hat{c}$ now represent the cavity fluctuations near the probe frequencies. We also use similar substitutions for the input field amplitudes, such that $\hat{a}_\mathrm{in}$ and $\hat{c}_\mathrm{in}$ are the input probe field operators. Neglecting small order terms, and accounting for a static mechanical shift induced by constant radiation pressure, our interaction Hamiltonian becomes

$$\hat{H}_\mathrm{int} = -\hbar g_\mathrm{a}\left(\alpha_\mathrm{a}\hat{a}^\dagger + \alpha_\mathrm{a}^*\hat{a}\right)\left(\hat{b} + \hat{b}^\dagger\right) - \hbar g_\mathrm{c}\left(\alpha_\mathrm{c}\hat{c}^\dagger + \alpha_\mathrm{c}^*\hat{c}\right)\left(\hat{b} + \hat{b}^\dagger\right). \tag{13}$$

We consider the case where the control lasers are red–detuned, with the probe fields on resonance such that $\Delta_\mathrm{i}^\mathrm{ctrl} = \omega_\mathrm{i}^\mathrm{ctrl} - \omega_\mathrm{i} = -\omega_\mathrm{b}$, and $\omega_\mathrm{i}^\mathrm{probe} - \omega_\mathrm{i}^\mathrm{ctrl} = \omega_\mathrm{b}$, where $i = \{\mathrm{a,c}\}$. In this case, selecting only the resonant terms under the rotating wave approximation, the above expression simplifies to

$$\hat{H}_\mathrm{int} = -\hbar\left(G_\mathrm{a}\hat{a}^\dagger\hat{b} + G_\mathrm{a}^*\hat{a}\hat{b}^\dagger + G_\mathrm{c}\hat{c}^\dagger\hat{b} + G_\mathrm{c}^*\hat{c}\hat{b}^\dagger\right), \tag{14}$$

where $G_\mathrm{a} = \alpha_\mathrm{a} g_\mathrm{a}$ and $G_\mathrm{c} = \alpha_\mathrm{c} g_\mathrm{c}$. Transforming into frequency space, in a frame rotating with the control lasers, and making use of Supplementary Equations (11-12) and (14) we may solve for the mode operators using the set of coupled linear equations

$$\begin{bmatrix} \chi_\mathrm{a}^{-1}(\omega) & -\mathrm{i}\,G_\mathrm{a} & 0 \\ -\mathrm{i}\,G_\mathrm{a}^* & \chi_\mathrm{b}^{-1}(\omega) & -\mathrm{i}\,G_\mathrm{c}^* \\ 0 & -\mathrm{i}\,G_\mathrm{c} & \chi_\mathrm{c}^{-1}(\omega) \end{bmatrix} \begin{bmatrix} \hat{a} \\ \hat{b} \\ \hat{c} \end{bmatrix} = \begin{bmatrix} \sqrt{\kappa_\mathrm{a}^\mathrm{ex}}\hat{a}_\mathrm{in} \\ \sqrt{\gamma_\mathrm{b}}\hat{b}_\mathrm{in} \\ \sqrt{\kappa_\mathrm{c}^\mathrm{ex}}\hat{c}_\mathrm{in} \end{bmatrix}. \tag{15}$$

In the above we have written the cavity susceptibilities as $\chi_\mathrm{a}^{-1}(\omega) = \kappa_\mathrm{a}/2 - \mathrm{i}(\Delta_\mathrm{a} + \omega)$, and $\chi_\mathrm{c}^{-1}(\omega) = \kappa_\mathrm{c}/2 - \mathrm{i}(\Delta_\mathrm{c} + \omega)$, where, for notational cleanliness we have defined $\Delta_\mathrm{a} = \Delta_\mathrm{a}^\mathrm{ctrl}$, and $\Delta_\mathrm{c} = \Delta_\mathrm{c}^\mathrm{ctrl}$. We also define the mechanical susceptibility as $\chi_\mathrm{b}^{-1}(\omega) = \gamma_\mathrm{b}/2 - \mathrm{i}(-\omega_\mathrm{b} + \omega)$, including a mechanical input field, $\hat{b}_\mathrm{in}$, which can be used to model thermal contact with the environment.

From here the solutions become tractable if we make a change of basis to symmetric and antisymmetric combinations of the a and c modes which we refer to as the mechanically dark, ζ_dk, and bright, ζ_br, modes[11]

$$\hat{\zeta}_\mathrm{dk} = \frac{G_\mathrm{c}\hat{a} - G_\mathrm{a}\hat{c}}{\mathrm{i}\,\overline{G}}, \tag{16}$$

$$\hat{\zeta}_\mathrm{br} = \frac{G_\mathrm{a}^*\hat{a} + G_\mathrm{c}^*\hat{c}}{\overline{G}}, \tag{17}$$

where

$$\overline{G} = \sqrt{|G_\mathrm{a}|^2 + |G_\mathrm{c}|^2}. \tag{18}$$

Assuming $\kappa_1 = \kappa_2 = \kappa$, and $\Delta_\mathrm{a} = \Delta_\mathrm{c} = \Delta$, we arrive at de–coupled equations of motion, which have the solutions

$$\hat{\zeta}_\mathrm{dk} = \frac{1}{\kappa/2 - \mathrm{i}(\Delta + \omega)}\left(\frac{\sqrt{\kappa_\mathrm{a}^\mathrm{ex}}G_\mathrm{c}\hat{a}_\mathrm{in} - \sqrt{\kappa_\mathrm{c}^\mathrm{ex}}G_\mathrm{a}\hat{c}_\mathrm{in}}{\mathrm{i}\,\overline{G}}\right), \tag{19}$$

$$\hat{b} = \frac{1}{\gamma_\mathrm{b}/2 - \mathrm{i}(-\omega_\mathrm{b} + \omega)}\left(\sqrt{\gamma_\mathrm{b}}\hat{b}_\mathrm{in} + \mathrm{i}\,\overline{G}\hat{\zeta}_\mathrm{br}\right), \tag{20}$$

$$\hat{\zeta}_\mathrm{br} = \frac{1}{\kappa/2 - \mathrm{i}(\Delta + \omega) + \frac{\overline{G}^2}{\gamma_\mathrm{b}/2 - \mathrm{i}(-\omega_\mathrm{b} + \omega)}}\left(\frac{\sqrt{\kappa_\mathrm{a}^\mathrm{ex}}G_\mathrm{a}^*\hat{a}_\mathrm{in} + \sqrt{\kappa_\mathrm{c}^\mathrm{ex}}G_\mathrm{c}^*\hat{c}_\mathrm{in}}{\overline{G}} + \frac{\mathrm{i}\,\overline{G}\sqrt{\gamma_\mathrm{b}}\hat{b}_\mathrm{in}}{\gamma_\mathrm{b}/2 - \mathrm{i}(-\omega_\mathrm{b} + \omega)}\right). \tag{21}$$

To easily access the physics of the system we take $G_\mathrm{a} = G_\mathrm{c} = G$, and $\kappa_\mathrm{a}^\mathrm{ex} = \kappa_\mathrm{c}^\mathrm{ex} = \kappa_\mathrm{ex}$ to simplify these expressions. We also ignore any input mechanical drive by setting $\hat{b}_\mathrm{in} \rightarrow 0$. Finally, we assume classical probe fields of equal amplitude, s_in, and drive each modulator at the same frequency with phase difference ϕ by making the substitutions $\hat{a}_\mathrm{in} \rightarrow s_\mathrm{in}e^{i\phi/2}$ and $\hat{c}_\mathrm{in} \rightarrow s_\mathrm{in}e^{-i\phi/2}$. This results in the expressions,

$$\zeta_\mathrm{dk} = \frac{\sqrt{2\kappa_\mathrm{ex}}\sin(\phi/2)s_\mathrm{in}}{\kappa/2 - \mathrm{i}(\Delta + \omega)}, \tag{22}$$

$$\zeta_\mathrm{br} = \frac{\sqrt{2\kappa_\mathrm{ex}}\cos(\phi/2)s_\mathrm{in}}{\kappa/2 - \mathrm{i}(\Delta + \omega) + \frac{2G^2}{\gamma_\mathrm{b}/2 - \mathrm{i}(-\omega_\mathrm{b} + \omega)}}. \tag{23}$$

Supplementary Note 5 - Effect of mismatched parameters

In the above expressions, we developed a model assuming idealized parameters. This resulted in mechanically bright and mechanically dark states which were decoupled from each other, and which could be isolated by adjusting the phase of the probe lasers. However, in any physical implementation of DOMIT, there will be mismatch between various parameters. In the following sections we study the effect of these mismatched parameters one by one.

Mismatched probe amplitudes

Suppose that all parameters are matched according to the set of assumptions the led to Supplementary Equations (22) and (23). We can include the effect of probe mismatch by instead making the substitutions $\hat{a}_{\text{in}} \to (s_{\text{in}} + \delta_{\text{s}})\, e^{i\phi/2}$ and $\hat{c}_{\text{in}} \to (s_{\text{in}} - \delta_{\text{s}})\, e^{-i\phi/2}$, where s_{in} is the average probe power, and $2\delta_{\text{s}}$ is the difference in the probe powers. Proceeding as before, we find,

$$\zeta_{\text{dk}} = \frac{\sqrt{2\kappa_{\text{ex}}}\,(\sin(\phi/2)s_{\text{in}} - i\cos(\phi/2)\delta_{\text{s}})}{\kappa/2 - \mathrm{i}(\Delta+\omega)}, \tag{24}$$

$$\zeta_{\text{br}} = \frac{\sqrt{2\kappa_{\text{ex}}}\,(\cos(\phi/2)s_{\text{in}} - i\sin(\phi/2)\delta_{\text{s}})}{\kappa/2 - \mathrm{i}(\Delta+\omega) + \frac{2G^2}{\gamma_{\text{b}}/2 - \mathrm{i}(-\omega_{\text{b}}+\omega)}}.. \tag{25}$$

From the above expression, one can see that for $\delta_{\text{s}} \neq 0$ no choice of ϕ will enable complete isolation of the dark or bright state. This is further elucidated by calculating the dependence of the mode energy on ϕ and δ_{s} for constant input probe power,

$$|\zeta_{\text{dk}}|^2 \propto \frac{\left(|s_{\text{in}}|^2 - |\delta_{\text{s}}|^2\right)\sin^2(\phi/2) + |\delta_{\text{s}}|^2}{|s_{\text{in}}|^2 + |\delta_{\text{s}}|^2}, \tag{26}$$

$$|\zeta_{\text{br}}|^2 \propto \frac{\left(|s_{\text{in}}|^2 - |\delta_{\text{s}}|^2\right)\cos^2(\phi/2) + |\delta_{\text{s}}|^2}{|s_{\text{in}}|^2 + |\delta_{\text{s}}|^2}. \tag{27}$$

Mismatched optomechanical coupling

The effect of mismatch in the optomechanical coupling will have similar effects to mismatch in the probe amplitudes. This can be included by making the substitutions $G_{\text{a}} \to G + \delta_{\text{G}}$ and $G_{\text{c}} \to G - \delta_{\text{G}}$, where G is the average optomechanical coupling rate, and $2\delta_{\text{G}}$ is the difference in optomechanical coupling rates. With these substitutions, the amplitudes of the mechanically bright and mechanically dark state are,

$$\zeta_{\text{dk}} = \frac{\sqrt{2\kappa_{\text{ex}}}s_{\text{in}}}{\kappa/2 - \mathrm{i}(\Delta+\omega)}\left(\frac{G\sin(\phi/2) + i\delta_{\text{G}}\cos(\phi/2)}{\overline{G}}\right), \tag{28}$$

$$\zeta_{\text{br}} = \frac{\sqrt{2\kappa_{\text{ex}}}s_{\text{in}}}{\kappa/2 - \mathrm{i}(\Delta+\omega) + \frac{2G^2}{\gamma_{\text{b}}/2 - \mathrm{i}(-\omega_{\text{b}}+\omega)}}\left(\frac{G\cos(\phi/2)s_{\text{in}} + i\delta_{\text{G}}\sin(\phi/2)}{\overline{G}}\right), \tag{29}$$

where $\overline{G} = \sqrt{G^2 + \delta_{\text{G}}^2}$ in this case. Calculating the mode amplitudes, we find,

$$|\zeta_{\text{dk}}|^2 \propto \frac{\left(|G|^2 - |\delta_{\text{G}}|^2\right)\sin^2(\phi/2) + |\delta_{\text{G}}|^2}{|\overline{G}|^2}, \tag{30}$$

$$|\zeta_{\text{br}}|^2 \propto \frac{\left(|G|^2 - |\delta_{\text{G}}|^2\right)\cos^2(\phi/2) + |\delta_{\text{G}}|^2}{|\overline{G}|^2}. \tag{31}$$

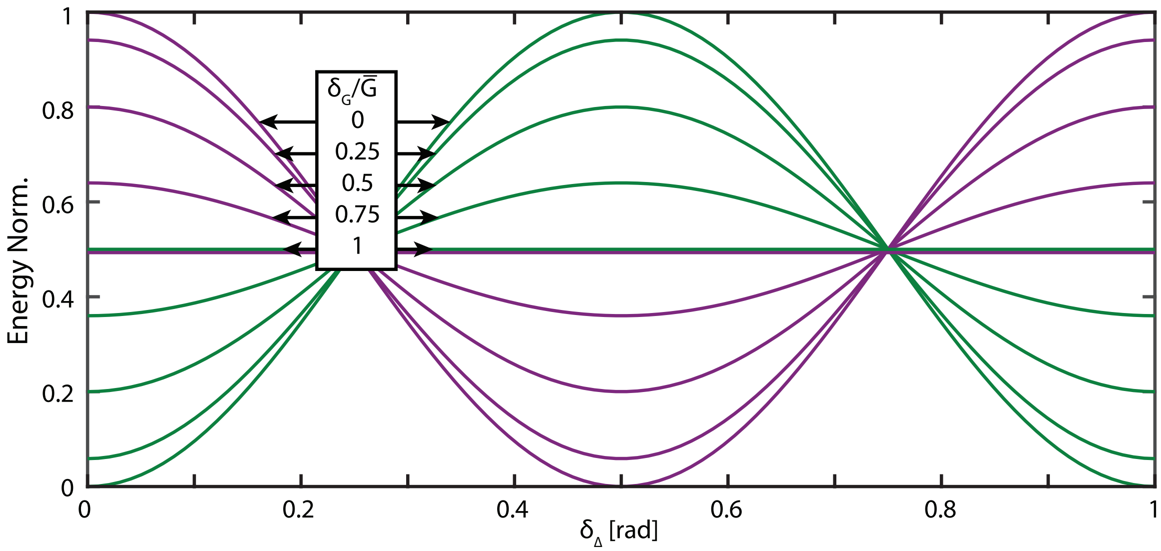

Supplementary Figure 4: Effect of imbalance in the probe powers, or optomechanical coupling rates on the amplitude of the bright and dark states. The amplitude of the dark (green) and bright (purple) state as a function of cavity-probe detuning, δ_Δ is reduced as the mismatch in optomechanical coupling rate, δ_G, increases.

Mismatched frequency and damping

Up until this point, we have found no direct coupling between the bright and dark mode. However, by detuning either our probe or pump lasers in equal and opposite directions, we can induce a coupling between these two modes. Furthermore, as we shall show, a mismatching the damping rates of the optical modes will also lead to a coupling. To see this, we make the substitutions $\Delta_\mathrm{a} \to \Delta + \delta_\Delta$, $\Delta_\mathrm{c} \to \Delta - \delta_\Delta$, $\kappa_\mathrm{a} \to \kappa + \delta_\kappa$, and $\kappa_\mathrm{c} \to \kappa - \delta_\kappa$. To clarify matters, we assume that the input mechanical is negligible ($\hat{b}_\mathrm{in} \to 0$), and set $G_\mathrm{a} = G_\mathrm{c} = G$, and $\kappa_1 = \kappa_2 = \kappa$ in Eqn. (4), which gives

$$\chi^{-1}(\omega)\hat{\zeta}_\mathrm{dk} = \frac{\sqrt{\kappa_\mathrm{a}^\mathrm{ex}}\hat{a}_\mathrm{in} - \sqrt{\kappa_\mathrm{c}^\mathrm{ex}}\hat{c}_\mathrm{in}}{\sqrt{2}\,\mathrm{i}} + \left(\delta_\Delta + i\frac{\delta_\kappa}{2}\right)\hat{\zeta}_\mathrm{br}, \tag{32}$$

$$\chi^{-1}(\omega)\hat{\zeta}_\mathrm{br} = \frac{\sqrt{\kappa_\mathrm{a}^\mathrm{ex}}\hat{a}_\mathrm{in} + \sqrt{\kappa_\mathrm{c}^\mathrm{ex}}\hat{c}_\mathrm{in}}{\sqrt{2}} + \mathrm{i}\,\overline{G}\hat{b} + \left(\delta_\Delta + i\frac{\delta_\kappa}{2}\right)\hat{\zeta}_\mathrm{dk}. \tag{33}$$

where $\chi^{-1}(\omega) = \kappa/2 - \mathrm{i}(\Delta + \omega)$. From these expressions we see that there is coupling between bright and dark mode. For differences in frequency we have dispersive coupling, at a rate δ_Δ, whereas for differences in damping, we have dissipative coupling at a rate $\delta_\kappa/2$.

Supplementary Note 6 - Dark-Bright mode coupling

In this section, we consider dissipative coupling due to detuning either our probe or pump lasers in equal and opposite directions. To describe this coupling in the time domain we first consider the intermodal coupling for the case $\delta_\Delta = 0$, as illustrated in Supplementary Figure 5(a). Here, depending on the relative phase of the probe lasers, we arrive at a superposition of ζ_br and ζ_dk which is constant in time. In order for this process to remain stationary, we require interference to be between oscillations of the same frequency. From this, we can infer that by shifting the probe-cavity detuning by an amount δ_Δ, we cause interference to occur between differing frequencies, leading

to beating between modes as illustrated in Supplementary Figure 5(b).

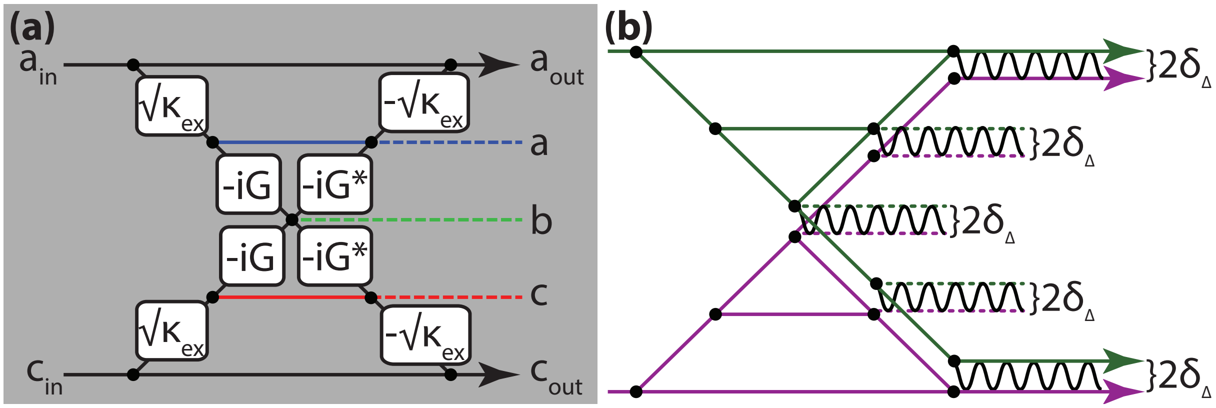

Supplementary Figure 5: Coupling the mechanically bright and dark states. (a) Intermodal coupling for the case of stationary DOMIT. Direction of propagation is left to right. (b) Effect of frequency shifting the control-probe detunings in opposite directions. Unlike the stationary case, we will observe a beating between all modes of the system.

To solve this, we divide each of modes into two frequency components at $\pm\delta_\Delta$ of our original frequency terms as illustrated in Supplementary Figure 2(b). Considering first the dark state in the time domain, and choosing to set $\phi = 0$ for convenience, we find

$$\zeta_{\rm dk}(t) = \zeta_{\rm dk}^{(+)} + \zeta_{\rm dk}^{(-)} = \frac{e^{-\,{\rm i}(\omega_{\rm b}+\delta_\Delta)t}}{\kappa/2 - {\rm i}(\Delta+\omega_{\rm b}+\delta_\Delta)}\sqrt{\frac{\kappa^{\rm ex}}{2}}\frac{s_{\rm in}}{\rm i} + \frac{e^{-\,{\rm i}(\omega_{\rm b}-\delta_\Delta)t}}{\kappa/2 - {\rm i}(\Delta+\omega_{\rm b}-\delta_\Delta)}\sqrt{\frac{\kappa^{\rm ex}}{2}}\frac{s_{\rm in}}{\rm i}. \tag{34}$$

In a similar manner, we find that the bright state may be written as

$$\begin{aligned}\zeta_{\rm br}(t) &= \zeta_{\rm br}^{(+)} + \zeta_{\rm br}^{(-)} \\ &= \frac{e^{-\,{\rm i}(\omega_{\rm b}+\delta_\Delta)t}}{\kappa/2 - {\rm i}(\Delta+\omega_{\rm b}+\delta_\Delta) + \frac{|\overline{G}|^2}{\gamma_{\rm b}/2 - {\rm i}(-\omega_{\rm b}+\omega_{\rm b}+\delta_\Delta)}}\sqrt{\frac{\kappa^{\rm ex}}{2}}s_{\rm in} \\ &\quad - \frac{e^{-\,{\rm i}(\omega_{\rm b}-\delta_\Delta)t}}{\kappa/2 - {\rm i}(\Delta+\omega_{\rm b}-\delta_\Delta) + \frac{|\overline{G}|^2}{\gamma_{\rm b}/2 - {\rm i}(-\omega_{\rm b}+\omega_{\rm b}-\delta_\Delta)}}\sqrt{\frac{\kappa^{\rm ex}}{2}}s_{\rm in}.\end{aligned} \tag{35}$$

Setting $\Delta = -\omega_{\rm b}$, and assuming $\delta \ll \kappa$, we find

$$\zeta_{\rm dk}(t) = \frac{2\sqrt{\kappa_{\rm ex}}\sin(\delta_\Delta t)e^{-\,{\rm i}\,\omega_{\rm b}t}s_{\rm in}}{\kappa}, \tag{36}$$

$$\zeta_{\rm br}(t) = \frac{2\sqrt{\kappa_{\rm ex}}\cos(\delta_\Delta t)e^{-\,{\rm i}\,\omega_{\rm b}t}s_{\rm in}}{\kappa(1+\frac{\overline{C}}{1+4(\delta_\Delta/\gamma_{\rm b})^2})}. \tag{37}$$

This gives us the output fields as

$$a(t) = \frac{e^{-\,{\rm i}\,\omega_{\rm b}t}}{\sqrt{2}}\left({\rm i}\sin(\delta_\Delta t)\zeta_{\rm dk}(0) + \cos(\delta_\Delta t)\zeta_{\rm br}(0)\right), \tag{38}$$

$$c(t) = \frac{e^{-\,{\rm i}\,\omega_{\rm b}t}}{\sqrt{2}}\left(-\,{\rm i}\sin(\delta_\Delta t)\zeta_{\rm dk}(0) + \cos(\delta_\Delta t)\zeta_{\rm br}(0)\right). \tag{39}$$

We note that near resonance, the amplitudes $\zeta_{\rm dk}$ and $\zeta_{\rm br}$ approach those calculated for steady state. For cases where we detuned away from the DOMIT transparency ($\delta \gg \gamma_{\rm b}$), or for small cooperativities, the amplitudes of the dark

and light state approach each other, and the visibility of oscillations goes to zero. Explicitly, the resonance contrast of the oscillations is found to be

$$V(\delta) = 1 - \sqrt{\frac{\gamma_{\mathrm{b}}^2 + 4\delta_{\Delta}^2}{\gamma_{\mathrm{b}}^2(1+\overline{C})^2 + 4\delta_{\Delta}^2}}. \tag{40}$$

Supplementary Note 7 - Switching

Although we previously found solutions in the frequency domain, it is instructive to reconsider the equations of motion in the time domain

$$\dot{\hat{a}} = (\mathrm{i}\,\Delta_{\mathrm{a}} - \kappa_{\mathrm{a}}/2)\,\hat{a} + \mathrm{i}\,G_{\mathrm{a}}\hat{b} + \sqrt{\kappa_{\mathrm{a}}^{\mathrm{ex}}}\hat{a}_{\mathrm{in}}, \tag{41}$$

$$\dot{\hat{b}} = (-\,\mathrm{i}\,\omega_{\mathrm{b}} - \gamma_{\mathrm{b}}/2)\,\hat{b} + \mathrm{i}\,G_{\mathrm{a}}^{*}\hat{a} + \mathrm{i}\,G_{\mathrm{c}}^{*}\hat{c} + \sqrt{\gamma_{\mathrm{b}}}\hat{b}_{\mathrm{in}}, \tag{42}$$

$$\dot{\hat{c}} = (\mathrm{i}\,\Delta_{\mathrm{c}} - \kappa_{\mathrm{c}}/2)\,\hat{c} + \mathrm{i}\,G_{\mathrm{c}}\hat{b} + \sqrt{\kappa_{\mathrm{c}}^{\mathrm{ex}}}\hat{c}_{\mathrm{in}}. \tag{43}$$

For the devices used in our experiment, the decay rate of our optics is much faster than our mechanics ($\kappa_{\mathrm{a}}, \kappa_{\mathrm{c}} \gg \gamma$). With this in mind we can use adiabatic elimination, and set $\dot{\hat{a}} = 0, \dot{\hat{c}} = 0$, and solve for the mechanics as

$$\dot{\hat{b}} = \left(-\,\mathrm{i}\,\omega_{\mathrm{b}} - \gamma_{\mathrm{b}}/2 + \frac{|G_{\mathrm{a}}|^2}{\mathrm{i}\,\Delta_{\mathrm{a}} - \kappa_{\mathrm{a}}/2} + \frac{|G_{\mathrm{c}}|^2}{\mathrm{i}\,\Delta_{\mathrm{c}} - \kappa_{\mathrm{c}}/2}\right)\hat{b} - \frac{\mathrm{i}\,G_{\mathrm{a}}^{*}\sqrt{\kappa_{\mathrm{a}}^{\mathrm{ex}}}\hat{a}_{\mathrm{in}}}{\mathrm{i}\,\Delta_{\mathrm{a}} - \kappa_{\mathrm{a}}/2} - \frac{\mathrm{i}\,G_{\mathrm{c}}^{*}\sqrt{\kappa_{\mathrm{c}}^{\mathrm{ex}}}\hat{c}_{\mathrm{in}}}{\mathrm{i}\,\Delta_{\mathrm{c}} - \kappa_{\mathrm{c}}/2} + \sqrt{\gamma_{\mathrm{b}}}\hat{b}_{\mathrm{in}}. \tag{44}$$

Using this expression we find that for $\Delta_{\mathrm{a}} = \Delta_{\mathrm{c}} = 0$, as in the experiment

$$\dot{\hat{b}} = -\left(\mathrm{i}\,\omega_{\mathrm{b}} + \tau^{-1}\right)\hat{b} + \frac{2\,\mathrm{i}\,G_{\mathrm{a}}\sqrt{\kappa_{\mathrm{a}}^{\mathrm{ex}}}\hat{a}_{\mathrm{in}}}{\kappa_{\mathrm{a}}} + \frac{2\,\mathrm{i}\,G_{\mathrm{c}}\sqrt{\kappa_{\mathrm{c}}^{\mathrm{ex}}}\hat{c}_{\mathrm{in}}}{\kappa_{\mathrm{c}}} + \sqrt{\gamma_{\mathrm{b}}}\hat{b}_{\mathrm{in}}. \tag{45}$$

This gives the switching speed as $\tau^{-1} = \frac{\gamma_{\mathrm{b}}}{2}\,(1 + C_{\mathrm{a}} + C_{\mathrm{c}})$, where $C_{\mathrm{j}} = 4G_{\mathrm{j}}^2/\kappa_{\mathrm{j}}\gamma_{\mathrm{b}}$ is the optomechanical cooperativity and $j = \{\mathrm{a}, \mathrm{c}\}$.

Assuming travelling wave singlet modes, the transmission amplitudes through the switch are

$$t_{\mathrm{br}} = \frac{2\kappa_{\mathrm{ex}}}{\kappa}\frac{1}{1+\overline{C}} - 1, \tag{46}$$

$$t_{\mathrm{dk}} = \frac{2\kappa_{\mathrm{ex}}}{\kappa} - 1. \tag{47}$$

Supplementary Note 8 - N-mode solution

Mechanically bright and dark states

The interference between probe fields in two optical modes can in principle be extended to any number of optical modes. Suppose we have a system where N optical modes are dissipatively coupled to a single mechanical mode. We label the creation and annihilation operators associated with these optical modes as $\hat{a}_n^{\dagger}, \hat{a}_n$ where our index runs from zero to $N-1$ and the mechanical mode creation and annihilation operators as is $\hat{b}^{\dagger}, \hat{b}$. We assume red detuned pumps, and that the system is sideband resolved. Our interaction Hamiltonian is:

$$\hat{H}_{\mathrm{int}} = -\hbar\sum_{n=0}^{N-1}\left(G_n\hat{a}_n^{\dagger}\hat{b} + G_n^{*}\hat{a}_n\hat{b}^{\dagger}\right). \tag{48}$$

Using the same set of assumptions as the DOMIT section above, we can use Supplementary Equation (48) and the input-output formalism to write a set of $N+1$ coupled equations,

$$\dot{\hat{a}}_n = \left(i\Delta_n - \frac{\kappa_n}{2}\right)\hat{a}_n + iG_n\hat{b} + \sqrt{\kappa_n^{\text{ex}}}\hat{a}_n^{\text{in}}, \tag{49}$$

$$\dot{\hat{b}} = \left(-i\omega_b - \frac{\Gamma_b}{2}\right)\hat{b} + i\sum_{n=0}^{N-1} G_n^*\hat{a}_n + \sqrt{\gamma_b}\hat{b}^{\text{in}}, \tag{50}$$

where we have indexed the decay rates for each mode to account for the possibility of mismatched decay rates.

This set of equations may be solved in a similar manner to DOMIT by seeking for a new basis where one mode is maximally coupled to the mechanics. To do so, we assume $\kappa_n = \kappa$, $\Delta_n = \Delta$, and $G_n = G$. Inspection of Supplementary Equation (48), reveals that the mechanics couples to the summation of all optical modes. Designating this as our mechanically bright mode, it then remains to construct a set of $N-1$ orthogonal modes. This may be achieved by applying a discrete Fourier transform to the optical modes,

$$\hat{\zeta}_m = \frac{1}{\sqrt{N}}\sum_{n=0}^{N-1}\hat{a}_n e^{-\frac{2\pi i}{N}nm}. \tag{51}$$

Here ζ_0 is the mechanically bright mode, and all others are mechanically dark modes. The optical input to the cavity is defined in the same manner,

$$\hat{\zeta}_m^{\text{in}} = \frac{1}{\sqrt{N}}\sum_{n=0}^{N-1}\hat{a}_n^{\text{in}} e^{-\frac{2\pi i}{N}nm}. \tag{52}$$

In this basis, our equations take on the simpler form,

$$\dot{\hat{\zeta}}_0 = \left(i\Delta - \frac{\kappa}{2}\right)\hat{\zeta}_0 + i\overline{G}\hat{b} + \sqrt{\kappa^{\text{ex}}}\hat{\zeta}_0^{\text{in}}, \tag{53}$$

$$\dot{\hat{b}} = \left(-i\omega_b - \frac{\gamma_b}{2}\right)\hat{b} + i\overline{G}\hat{\zeta}_0 + \sqrt{\gamma_b}\hat{b}^{\text{in}}, \tag{54}$$

$$\dot{\hat{\zeta}}_m = \left(i\Delta - \frac{\kappa}{2}\right)\hat{\zeta}_m + \sqrt{\kappa^{\text{ex}}}\hat{\zeta}_m^{\text{in}}. \tag{55}$$

Where in the above the m index in the ζ_m equations runs from 1 to N, and $\overline{G} = \sqrt{N}G$.

These equations can be solved by transforming into frequency space,

$$\hat{\zeta}_0(\omega) = \frac{1}{\kappa/2 - i(\omega+\Delta) + \frac{N|G|^2}{\gamma_b/2 - i(\omega-\omega_b)}}\left(\sqrt{\kappa^{\text{ex}}}\hat{\zeta}_0^{\text{in}} + \frac{i\sqrt{\gamma_b}\overline{G}\hat{b}^{\text{in}}}{\gamma_b/2 - i(\omega-\omega_b)}\right), \tag{56}$$

$$\hat{b}(\omega) = \frac{1}{\gamma/2 - i(\omega-\omega_{\text{b}}) + \frac{N|G|^2}{\kappa/2 - i(\omega+\Delta)}}\left(\sqrt{\gamma_b}\hat{b}^{\text{in}} + \frac{i\sqrt{\kappa^{\text{ex}}}\,\overline{G}\hat{\zeta}_0^{\text{in}}}{\kappa/2 - i(\omega+\Delta)}\right), \tag{57}$$

$$\zeta_m(\omega) = \frac{\sqrt{\kappa^{\text{ex}}}\hat{\zeta}_m^{\text{in}}}{\kappa/2 - i(\omega+\Delta)}. \tag{58}$$

To gain insight into these expressions, we consider the on resonance ($\Delta = -\omega_{\text{b}}, \omega = \omega_{\text{b}}$). This yields the expressions

$$\hat{\zeta}_0(\omega) = \frac{2/\kappa}{1+\overline{C}}\left(\sqrt{\kappa^{\text{ex}}}\hat{\zeta}_0^{\text{in}} + \frac{i2\overline{G}\hat{b}^{\text{in}}}{\sqrt{\gamma_{\text{b}}}}\right), \tag{59}$$

$$\hat{b}(\omega) = \frac{2/\gamma}{1+\overline{C}}\left(\sqrt{\gamma_{\text{b}}}\hat{b}^{\text{in}} + \frac{i2\overline{G}\zeta_0^{\text{in}}}{\kappa/\sqrt{\kappa^{\text{ex}}}}\right), \tag{60}$$

$$\zeta_m(\omega) = 2\sqrt{\kappa^{\text{ex}}}/\kappa\hat{\zeta}_m^{\text{in}}. \tag{61}$$

From these expressions it can be seen that optomechanical coupling is only present between the mechanics and the mechanically bright mode. All other optical modes will see a bare cavity response. Initially, as the optomechanical coupling is increased, the degree of exchange between the bright mode and the mechanics will also increase. This situation has some resemblance to an add-drop filter. For very large cooperativities, both the mechanics and the bright mode will suppressed, and the system acts as notch filter for these modes.

Interestingly, this general case subsumes many well studied optomechanical effects. For example, in the case of a single optical mode, only the bright state can exist. In this case the filtering effect describes OMIT, where the occupation of the optical cavity is suppressed. In the case of two optical modes, as discussed previously in this paper, both a mechanically bright and a dark mode may exist. In this case, selection of the mechanically dark mode can lead to coupling between different colours of input light, while avoiding decoherence due to the mechanics. The analysis here indicates that for larger dimensions, there will always exist $N-1$ such mechanically dark modes, which can avoid decoherence from the mechanics.

Outputs

Here we will calculate the output in the more physical basis of the individual optical modes. If we assume that b^{in} can be neglected, we can write out solutions in the simple form $\zeta_0 = \eta_0 \zeta_0^{\text{in}}$ for $m = 0$, and $\zeta_m = \eta_1 \zeta_m^{\text{in}}$ otherwise. Next we would like to return to our original basis in order to calculate the transmission at the physical ports. To do this, we use the inverse discrete Fourier transform, defined as

$$\hat{a}_n = \frac{1}{\sqrt{N}} \sum_{m=0}^{N-1} \hat{\zeta}_m e^{\frac{2\pi i}{N} nm}. \tag{62}$$

Placing our solutions into this expression we find

$$\begin{aligned} a_n =& \frac{\eta_0}{\sqrt{N}} \zeta_0^{\text{in}} + \frac{\eta_1}{\sqrt{N}} \sum_{m=1}^{N-1} \zeta_m^{\text{in}} e^{\frac{2\pi i}{N} nm}, \\ =& \frac{\eta_0 - \eta_1}{N} \sum_{n'=0}^{N-1} a_{n'}^{in} + \frac{\eta_1}{N} \sum_{n'=0}^{N-1} \left(\sum_{m=0}^{N-1} e^{\frac{-2\pi i}{N} m(n'-n)} \right) a_{n'}^{\text{in}}, \\ =& \frac{\eta_0 - \eta_1}{N} \sum_{n'=0}^{N-1} a_{n'}^{in} + \frac{\eta_1}{N} \sum_{n'=0}^{N-1} N \delta_{n'n} a_{n'}^{\text{in}}, \\ =& \frac{\eta_0 - \eta_1}{N} \sum_{n'=0}^{N-1} a_{n'}^{in} + \eta_1 a_n. \end{aligned} \tag{63}$$

If we take the on-resonance case, $\eta_0 = \frac{2\sqrt{\kappa^{\text{ex}}}}{\kappa}$ and $\eta_1 = \frac{2\sqrt{\kappa^{\text{ex}}}}{\kappa} \frac{1}{1+\overline{C}}$ where $\overline{C} = NC = \frac{4N|G|^2}{\kappa \Gamma_b}$. This allows us to write,

$$a_n = \frac{2\sqrt{\kappa^{\text{ex}}}}{\kappa} \left(a_n^{\text{in}} - \frac{\overline{C}}{N(1+\overline{C})} \sum_{n'=0}^{N-1} a_{n'}^{\text{in}} \right), \tag{64}$$

$$a_n^{\text{out}} = \left(1 - \frac{2\kappa^{\text{ex}}}{\kappa} a_n^{\text{in}} \right) + \frac{2\kappa^{\text{ex}}}{\kappa} \frac{\overline{C}}{N(1+\overline{C})} \sum_{n'=0}^{N-1} a_{n'}^{\text{in}}. \tag{65}$$

For the case of critical coupling, and large cooperativity,

$$a_n^{\text{out}} = -a_n^{\text{in}} + \frac{2}{N} \sum_{n'=0}^{N-1} a_{n'}^{\text{in}}. \tag{66}$$

This can be cast in matrix form as,

$$\begin{pmatrix} a_0^{\mathrm{out}} \\ a_1^{\mathrm{out}} \\ \vdots \\ a_{N-1}^{\mathrm{out}} \end{pmatrix} = \frac{1}{N} \begin{pmatrix} 2-N & 2 & \cdots & 2 \\ 2 & 2-N & \cdots & 2 \\ \vdots & \vdots & \ddots & \vdots \\ 2 & 2 & \cdots & 2-N \end{pmatrix} \begin{pmatrix} a_0^{\mathrm{in}} \\ a_1^{\mathrm{in}} \\ \vdots \\ a_{N-1}^{\mathrm{in}} \end{pmatrix}. \tag{67}$$

This indicates that complete conversion from one colour to another is only possible for the case $N = 2$.

Supplementary References

[1] Khanaliloo, B., Mitchell, M., Hryciw, A. C. & Barclay, P. E. High-Q/V monolithic diamond microdisks fabricated with quasi-isotropic etching. *Nano Lett.* **15**, 5131–5136 (2015).

[2] Mitchell, M., Lake, D. P. & Barclay, P. E. Realizing $Q >$ 300 000 in diamond microdisks for optomechanics via etch optimization. *APL Photonics* **4**, 016101 (2019).

[3] Lake, D. P., Mitchell, M., Kamaliddin, Y. & Barclay, P. E. Optomechanically induced transparency and cooling in thermally stable diamond microcavities. *ACS Photonics* **5**, 782–787 (2018).

[4] Aharonovich, I., Greentree, A. D. & Prawer, S. Diamond photonics. *Nat. Photonics* **5**, 397–405 (2011).

[5] Mitchell, M. *et al.* Single-crystal diamond low-dissipation cavity optomechanics. *Optica* **3**, 963–970 (2016).

[6] Kippenberg, T. J., Rokhsari, H., Carmon, T., Scherer, A. & Vahala, K. J. Analysis of radiation-pressure induced mechanical oscillation of an optical microcavity. *Phys. Rev. Lett.* **95**, 033901 (2005).

[7] Carmon, T., Rokhsari, H., Yang, L., Kippenberg, T. J. & Vahala, K. J. Temporal behavior of radiation-pressure-induced vibrations of an optical microcavity phonon mode. *Phys. Rev. Lett.* **94**, 223902 (2005).

[8] Rokhsari, H., Kippenberg, T. J., Carmon, T. & Vahala, K. J. Radiation-pressure-driven micro-mechanical oscillator. *Opt. Express* **13**, 5293–5301 (2005).

[9] Kippenberg, T. J., Spillane, S. M. & Vahala, K. J. Modal coupling in traveling-wave resonators. *Opt. Lett.* **27**, 1669–1671 (2002).

[10] Yan, L.-S., Yu, Q., Willner, A. E. & Shi, Y. Measurement of the chirp parameter of electro-optic modulators by comparison of the phase between two sidebands. *Opt. Lett.* **28**, 1114–1116 (2003).

[11] Dong, C., Fiore, V., Kuzyk, M. C. & Wang, H. Optomechanical dark mode. *Science* **338**, 1609–1613 (2012).
