# Supplementary material for: Two-colour interferometry and switching through optomechanical dark mode excitation
Source: Nat Commun. 2020 May 5;11:2208. doi: 10.1038/s41467-020-15625-x (PMC7200651; doi:10.1038/s41467-020-15625-x)
Supplement: Supplementary file 2 — Peer Review File [file 41467_2020_15625_MOESM2_ESM.pdf]

Editorial Note: This manuscript has been previously reviewed at another journal that is not operating a transparent peer review scheme. This document only contains reviewer comments and rebuttal letters for versions considered at Nature Communications.

REVIEWERS' COMMENTS:

Reviewer #1 (Remarks to the Author):

The authors have adequately addressed my questions and comments in the earlier report. The manuscript presents a nice experimental demonstration on phase-dependent switching between bright and dark optical modes in a multi-mode optomechanical system. The results reported can have potential applications in two-color optical switching as well as interferometry. I recommend the publication of the manuscript in Nature Communications.

Minor correction: Ref. 30 and Ref. 37 are the same.

Reviewer #2 (Remarks to the Author):

I have reread in detail the author's revised manuscript. I am satisfied with their responses to my original concerns and think they responded thoughtfully to all the reviewers' issues. I think this work is suitable for publication in Nature Communications.
